# Clonal Expansion of the *Pseudogymnoascus destructans* Genotype in North America Is Accompanied by Significant Variation in Phenotypic Expression

**DOI:** 10.1371/journal.pone.0104684

**Published:** 2014-08-14

**Authors:** Jordan Khankhet, Karen J. Vanderwolf, Donald F. McAlpine, Scott McBurney, David P. Overy, Durda Slavic, Jianping Xu

**Affiliations:** 1 Department of Biology, McMaster University, Hamilton, Ontario, Canada; 2 New Brunswick Museum, Saint John, New Brunswick, Canada; 3 Canadian Wildlife Federation, Kanata, Ontario, Canada; 4 Canadian Cooperative Wildlife Health Centre, Atlantic Region, Department of Pathology and Microbiology, Atlantic Veterinary College, University of Prince Edward Island, Charlottetown, Prince Edward Island, Canada; 5 Department of Chemistry, University of Prince Edward Island, Charlottetown, Prince Edward Island, Canada; 6 Department of Pathology and Microbiology, Atlantic Veterinary College, University of Prince Edward Island, Charlottetown, Prince Edward Island, Canada; 7 Nautilus Biosciences Canada Inc., Duffy Research Center, University of Prince Edward Island, Charlottetown, Prince Edward Island, Canada; 8 Laboratory Services, Animal Health Laboratory, University of Guelph, Guelph, Ontario, Canada; California Department of Public Health, United States of America

## Abstract

*Pseudogymnoascus destructans* is the causative agent of an emerging infectious disease that threatens populations of several North American bat species. The fungal disease was first observed in 2006 and has since caused the death of nearly six million bats. The disease, commonly known as white-nose syndrome, is characterized by a cutaneous infection with *P. destructans* causing erosions and ulcers in the skin of nose, ears and/or wings of bats. Previous studies based on sequences from eight loci have found that isolates of *P. destructans* from bats in the US all belong to one multilocus genotype. Using the same multilocus sequence typing method, we found that isolates from eastern and central Canada also had the same genotype as those from the US, consistent with the clonal expansion of *P. destructans* into Canada. However, our PCR fingerprinting revealed that among the 112 North American isolates we analyzed, three, all from Canada, showed minor genetic variation. Furthermore, we found significant variations among isolates in mycelial growth rate; the production of mycelial exudates; and pigment production and diffusion into agar media. These phenotypic differences were influenced by culture medium and incubation temperature, indicating significant variation in environmental condition - dependent phenotypic expression among isolates of the clonal *P. destructans* genotype in North America.

## Introduction

White-nose syndrome (WNS) is an emerging infectious disease that first appeared in New York in 2006. Since its emergence it has been estimated to have caused the death of over 5.5 million bats in North America [1], [2]. The causative agent of WNS is a psychrophilic fungus, *Pseudogymnoascus destructans* (formerly *Geomyces destuctans*) [3], [4]. WNS is characterized by the growth of white, fluffy, mycelia of *P. destructans* on the muzzle, wings and ears of hibernating bats, with the hyphae causing erosions and ulcers in the skin of bats and damaging the underlying subcutaneous tissue [5]. The recent rapid spread of WNS has led to the dire prediction that North America’s most abundant bat species, the little brown bat (*Myotis lucifugus*), may face regional extinction within two decades [6]. WNS has now been diagnosed in 25 US states and five Canadian provinces (http://www.whitenosesyndrome.org/resources/map). *Pseudogymnoascus destructans* is also widespread across Europe [7] although the infection is not fatal to European bats [8]. In contrast, infection by both European and North American sourced isolates of *P. destructans* are fatal to the North American little brown bat [9].

Previous studies have used multilocus sequence typing (MLST) and PCR fingerprinting to genotype *P. destructans* isolates from the US. No sequence variation was found among the samples examined and all isolates shared identical PCR fingerprints [10]–[12]. The apparent clonal genotype of the US population of *P. destructans* is consistent with the hypothesis that a single strain of *P. destructans* was recently introduced into the US. Given the timeline of case reports of WNS in Canada [13], [14], we hypothesize that Canadian occurrences of *P. destructans* represent the same clonal genotype as that in the US. However, whether the same clonal genotype is responsible for the widespread mortality of bats in Canada remains unconfirmed.

Aside from investigations of genotypic variation, several studies have examined how environmental factors influence the phenotypes of *P. destructans*. For example, Martinkova et al. (2010) showed that *P. destructans* could grow on a variety of media and that different media supported different rates of mycelial growth [15]. Verant et al. (2012) examined incubation temperature and found that it has a significant influence on mycelial growth of *P. destructans*, with optimal growth temperature range between 12.5 and 15.8°C [16]. Verant et al. (2012) also analyzed the growth responses to a variety of temperatures among six independent isolates, three from the US and three from Europe. They found that geographical origin did not influence the growth rate of *P. destructans* on artificial media and that all isolates showed significant responses to small changes in temperature [16]. At present, relatively little is known about the potential phenotypic variations within and among isolates of *P. destructans* from either North America or Europe in their response to differences in growth conditions.

Understanding the genotypic and phenotypic variation of pathogens could help us better track the spread of infectious diseases and develop strategies to prevent and control these diseases. The objectives of this study are to determine whether isolates of *P. destructans* from Canada are members of the same genotype as those from the US, and to examine the variations of four phenotypes (mycelial growth rate, pigment production, pigment secretion and diffusion, and exudate production) at various temperatures and on a range of media, among a set of geographically diverse North American isolates. While the role of the phenotypic traits we examined are not presently known in *P. destructans*, in other fungi these traits have been associated with vegetative fitness [17], fungal defense against the host immune system [18], and the production of fungal toxins and virulence factors in exudates [19], [20]. We hypothesized that if the *P. destructans* population in North America occurred via the recent clonal expansion of a single genotype, then there should be little/no genotypic and phenotype variation among the isolates.

## Materials and Methods

### Fungal Isolates

A total of 112 *P. destructans* isolates collected from 2008 to 2013 are included in this study (Table S1). The isolates were provided by the Animal Health Laboratory at the University of Guelph (Guelph, Ontario), the New Brunswick Museum (Saint John, New Brunswick), the Canadian Cooperative Wildlife Health Centre (Atlantic Region), and the Mycology Laboratory at Wadsworth Center in Albany, New York. One sample was also isolated at McMaster University by the first author of the study. In total, these isolates originated from four Canadian provinces; Ontario (16 isolates), New Brunswick (71 isolates), Nova Scotia (1 isolate), and Prince Edward Island (9 isolates) and six US states; New York (3 isolates), North Carolina (3 isolates), West Virginia (5 isolates), Ohio (1 isolate), Vermont (2 isolates), and Pennsylvania (1 isolate). The New Brunswick isolates originated from live bats, cave associated arthropods, and hibernaculum walls at seven hibernaculum sites. Isolates from Nova Scotia, Prince Edward Island, Ontario and the US originated from deceased bats confirmed to have died of WNS. All isolates from dead bats were from materials sent to the above respective laboratories from either the Natural Resources Departments in the various provinces in Canada where the bats originated from or the Fish and Wildlife Services (in US). Isolates were obtained by culturing swabs from various substrates and locations onto agar media. The mycelial colonies were then purified by repeated sub-culturing using hyphal tip cultures until a stable, morphologically uniform and pure colony was obtained for each isolate. The permissions for acquiring the materials for isolating *P. destructans* were granted by the Departments of Natural Resources in New Brunswick, Nova Scotia, Prince Edward Island, and Ontario (for Canadian isolates) and the US Fish and Wildlife Services (for US isolates).

### DNA Fingerprinting

Previous genotype analyses of isolates of *P. destructans* have used both multilocus sequence typing (MLST) and PCR fingerprinting [10]–[12]. PCR fingerprinting, based on changes in repetitive sequences, is easier to perform than MLST and the genomic regions sampled using this method are likely evolving faster than simple nucleotide substitutions [21]. In fungi, two separate primers targeting repetitive elements in the genome have been used extensively for distinguishing closely related strains by PCR fingerprinting. We followed this strategy, using primer (GACA)_4_ (5′GACAGACAGACAGACA-3′) and the M13-phage core sequence (5′-GAGGGTGGCGGTTCT-3′) to fingerprint isolates of *P. destructans* obtained from eastern and central Canada and compared them with representative isolates from the US.

*P. destructans* isolates were first grown on Sabouraud dextrose agar (SDA) plates and genomic DNA was extracted following the protocol outlined in Xu *et al.* (2000). For PCR fingerprinting, each PCR reaction contained 12 µL of Ready-to-Go PCR mix (Promega, Madison, WI, USA), 2 µL of template DNA, and 2 µL of 10 µM of primer. PCR conditions were as follows: 5-minute initial denaturation at 94°C, 35 amplification cycles with a 1 minute denaturation at 94°C, 1 minute annealing at 50°C, and 2-minute extension at 72°C, and an 8-minute final extension at 72°C. PCR amplicons were separated on a 1.25% agarose gel stained with ethidium bromide. Gel electrophoresis was run for 1 hour and 50 minutes at 150 V in TBE buffer and visualized under UV light using a Chemi-Imager (Alpha InnovTech Corporation, San Leandro, California). PCR fingerprints were scored independently by two investigators and isolates showing identical patterns by both investigators were considered to have the same genotype. Isolates shown to have fingerprint patterns different from the majority were analysed a second time to confirm their distinctiveness. The finalized fingerprint data were used to generate the relationship among isolates using the program PAUP 4.0 [22].

### Multilocus Sequence Typing

To confirm genotypes and compare the Canadian isolates with those obtained from the US, 44 of the 112 isolates representing a variety of bat hibernacula, a range of geographic locations, and all distinct PCR fingerprint genotypes were selected for multilocus genotyping, following protocols of Chaturvedi et al. (2010) [12]. The 44 isolates subjected to multilocus sequence typing are highlighted in Table S1. Specifically, the following eight gene fragments were amplified and sequenced and compared to those from the US: *ALR*, *Bpntase*, *DHC1*, *GPHN*, *PCS*, *POB3, SRP72*, and *VPS13* [10]. PCR reactions contained 13.6 µL of Ready-to-Go PCR mix (Promega, Madison, WI, USA), 2 µL of template DNA, and 0.4 µL of 10 µM of primer. The PCR conditions were as follows: 3-minute initial denaturation at 94°C, 40 amplification cycles with a 15-second denaturation at 94°C, 30-second annealing at 55°C, and 1-minute extension at 68°C and a 5-minute final extension at 68°C. For confirmation of successful PCR amplification, 5 µL of the amplified product for each gene from each sample was separated on a 1% agarose gel with ethidium bromide in TAE buffer for 25 minutes at 150 V. Successfully amplified PCR products were purified prior to sequencing using MicroClean©. Sequences were aligned in MEGA 5 [23] and genetic relationship among the strain was identified using maximum parsimony based on the concatenated sequences of the eight genes with a total of 4,573 nucleotides in length.

### Morphological Comparisons

To identify potential phenotypic variations within and among isolates and determine whether different isolates respond similarly to the same set of environmental variables, 16 isolates representing all genotypes and a range of geographic jurisdictions in Canada (Ontario, New Brunswick, Prince Edward Island and Nova Scotia) and the US (New York and North Carolina) were examined. Potential differences in growth and colony morphology on SDA, potato dextrose agar (PDA), and minimal media agar (MMA) at temperatures of 4°C, 14°C and 18°C were investigated. The SDA contained 1% enzymatic digest of casein (Difco), 2% dextrose and 2% agar (pH 5.6±0.2); PDA contained 0.4% potato starch (Difco), 2% dextrose and 1.5% agar (pH 5.6±0.2); and the MMA contained 0.17% yeast nitrogen base without amino acids and ammonium sulphate (Difco), 0.5% (NH_4_)_2_SO_4_, 2% dextrose and 2% agar (pH ∼6.0). Prior to culturing the isolates to determine phenotypic variations, we first cultured all isolates under the same condition (PDA/14°C) to set all isolates’ gene expression pattern to the same environment and to minimize potential influences of original isolating conditions on gene expression. Each isolate-medium-temperature combination had three replicates. Colony diameter was measured 28 days after incubation. Qualitative morphological features of fungal colonies were scored visually by the naked eye for three phenotypic traits: colony reverse color, pigment secretion and diffusion into surrounding media, and exudate production, all at 28 days after incubation. Pigment secretion and diffusion into surrounding media was scored as either present or absent. Colony reverse color was recorded as the color of the agar medium directly underneath each colony. Exudate production was scored on a scale of 0–3, with 0 = no exudate, 1 = little exudate, 2 = intermediate exudate, and 3 = profuse exudate.

### Statistical Analysis

Statistical analyses were performed for the quantitative data of mycelial growth rate using SPSS 20.0 (IBM, Chicago, IL). The colony diameters were analyzed using a three-way factorial analysis of variance (ANOVA). The factors were media, temperature and isolates. P values of <0.05 were considered statistically significant. One-way ANOVAs with the Games-Howell test or the Tukey’s HSD post hoc test were conducted to compare diameters of *P. destructans* isolates in different temperature-medium combinations. Tukey’s HSD test was used in conditions when the variances among the isolates were similar to each other while the Games-Howell test was used in conditions with unequal variances. The Bartlett’s test was used to examine the evenness of the variances within each of the nine treatments.

## Results

### Genotypes

**Figure 1:**
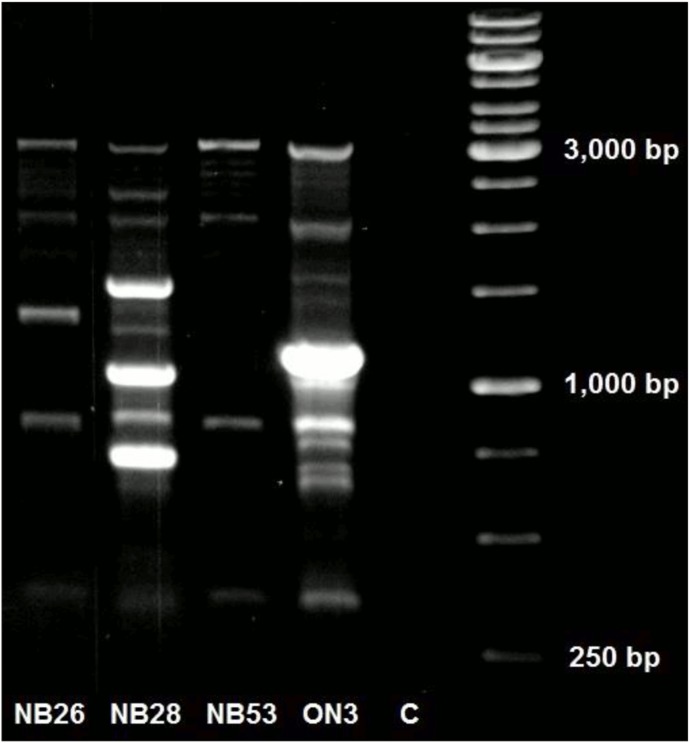
PCR fingerprint banding patterns of four *Pseudogymnoascus destructans* isolates representing four different genotypes using the M13 primer. Three distinct fingerprints are represented by NB26, NB28 and ON3. NB53 represents the dominant fingerprint which included 109 of the 112 isolates. Lane C is the negative control with distilled H_2_0 as template.

A total of 13 fragments ranging between 300–3000 bp were scored and the DNA fingerprinting data based on the two PCR primers revealed four distinct PCR fingerprints among the 112 *P. destructans* isolates (Fig. 1, Fig. S1). Three of these fingerprints were represented by a single isolate each, while the remaining 109 isolates belonged to the fourth genotype. The three unique isolates included one from Ontario (ON3) and two from New Brunswick (NB26- Dorchester Mine and NB28- Harbell’s Cave) (Fig. 1). ON3 was cultured from a deceased bat, NB26 from the hibernaculum wall and NB28 from a live bat. However, when these three isolates and other 41 representative isolates were subjected to MLST, all 44 isolates from eastern and central Canada (Ontario, New Brunswick, Prince Edward Island, Nova Scotia) were found to have the same MLST genotype identical to those from the US at the eight sequenced loci [10], [11]. Our results are thus consistent with the hypothesis of clonal expansion of *P. destructans* from the US to Canada, accompanied by genotypic microevolution.

### Mycelial Growth

Variation in mycelial growth was found among the 16 isolates on all nine media-temperature combinations. The ANOVA revealed that the isolate, medium and temperature all contributed significantly to the variation in colony diameters (p<0.05; Table 1). The three factors also interacted significantly in all three two-way combinations as well as the one three-way combination to influence variation in colony diameter (p<0.05; Table 1). The mean colony diameter and the standard deviation for each of the 16 *P. destructans* isolates at 28 days after inoculation are shown in Table S2. Of the three temperatures tested, 14°C was found to be the most conducive for mycelial growth for all 16 isolates tested on all three media. On two of the three media (MMA and SDA), 4°C was the next most favourable incubation temperature for mycelial growth followed by 18°C. However, on PDA, the reverse was observed.

**Table 1:** Three-way ANOVA for isolate-temperature-medium interactions on colony diameter for 16 North American *Pseudogymnoascus destructans* isolates 28 days after inoculation.

Source	df	SS	MS	F
Isolate (I)	15	1104.078	73.605	58.078***
Medium (M)	2	1584.962	792.481	625.300***
Temperature (T)	2	5143.753	2571.877	2029.316***
M×T	4	391.764	97.941	77.279***
M×I	30	99.983	3.333	2.630***
T×I	30	262.247	8.742	6.897***
M×T×I	58	174.292	2.905	2.292***
Error	278	365.000	1.267	

df, degrees of freedom; SS, sum of squares; MS, mean squares.

***p<0.001.

Interestingly, among the three tested temperatures, the most conducive for growth (14°C) also showed the largest range of colony diameters among the isolates. The two isolates with the smallest colony diameters, US3 and NS1, were consistently the smallest relative to the other isolates across all environmental conditions, with the exception of PDA/4°C. However, no single isolate had the largest colony diameter across all nine conditions. Pairwise comparisons revealed significant differences in colony diameters between isolates, for all nine conditions (Table S2). The SDA/4°C environment showed the largest number of significantly different colony diameters, with 34 pairs. MMA/14°C and SDA/14°C followed closely, with 30 and 28 pairs respectively. Taken together, our results indicate significant divergence in mycelial growth rate among asexual isolates recently derived from a single ancestor genotype. As mentioned, the observed growth rate variation was influenced by both media and temperature.

### Exudate Production

**Figure 2:**
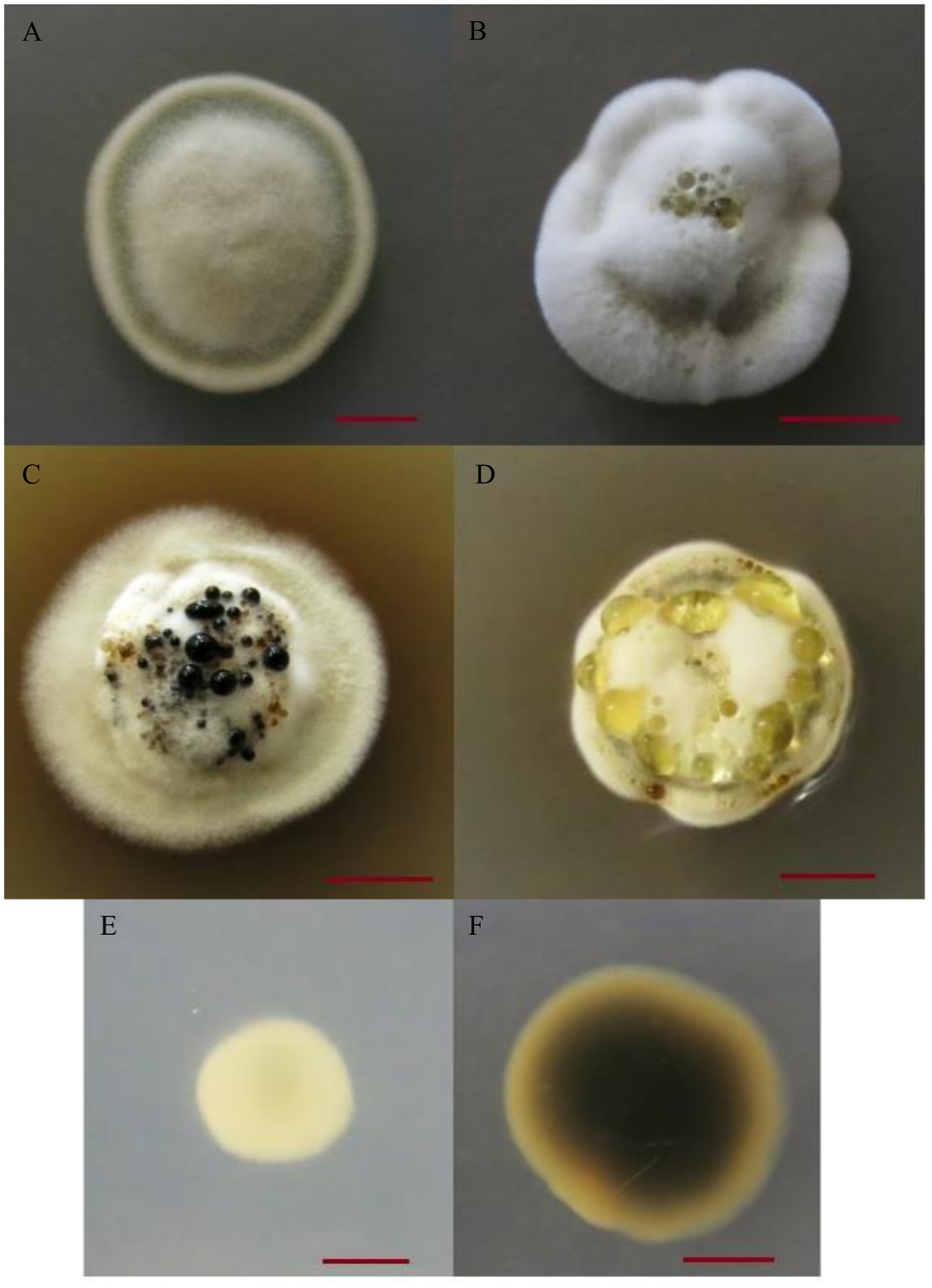
Morphological characteristics of *Pseudogymnoascus destructans* isolates following 28 days of incubation. (A) Typical isolate colony morphology on PDA/14°C represented by NB7. A score of 0 for exudate production was given. (B) Colony morphology of NB28 on SDA/4°C with a score of 1 for exudate production. (C) Colony morphology of ON16 on SDA 14°C. A score of 2 was given for exudate production and pigment secretion into medium was recorded. (D) Colony morphology of PE1 on PDA/18°C with a score of 3 for exudate production. (E) White reverse colony color of NB8 on MMA/4°C. (F) Black reverse colony color for ON16 on PDA/14°C. Scale bar indicates 5 mm. SDA = Sabouraud dextrose agar, PDA = potato dextrose agar, MMA = minimal medium agar.

**Figure 3:**
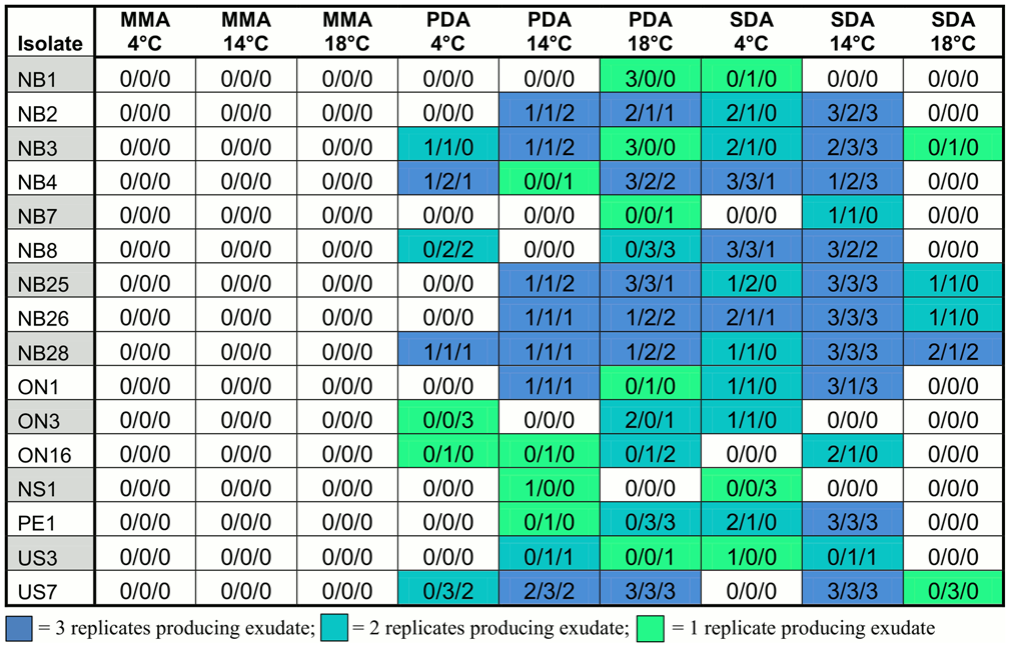
Variations in exudate production within and among 16 North American *Pseudogymnoascus destructans* isolates in nine conditions. 0 = no exudate production; 1 = small but visible exudate production; 2 = intermediate exudate production; 3 = profuse exudate production (for representative figures, please see Figure 2B–D). SDA = Sabouraud dextrose agar, PDA = potato dextrose agar, MMA = minimal medium agar. Three replicates are shown for each medium-temperature condition for each isolate.

Variation in exudate production was found among the 16 *P. destructans* isolates. All three factors (temperature, medium and isolate) contributed to the observed variation in exudate production. Representatives of scores 0–3 for exudate production are shown in Figures 2A–D. After 28 days of incubation, no exudate was observed on colonies cultured on MM at any of the three incubation temperatures for any of the 16 isolates. In contrast, exudates were often observed among isolates cultured on the two other media and each of the 16 isolates produced exudate in at least two of the six remaining conditions (Fig. 3). Significant differences in exudate production were observed among the isolates within each medium/temperature condition (Fig. 3). On SDA medium, 13 isolates produced exudates in at least one of the three replicates at 4°C and a slightly different combination of 13 isolates produced exudates at 14°C, while only 5 isolates produced exudates at 18°C (Fig. 3). Four isolates produced exudates at all three temperatures on SDA medium. On PDA, 7, 12, and 15 isolates produced exudates at 4°C, 14°C, and 18°C respectively and only 5 isolates produced exudates at all three temperatures (Fig. 3). NB3 and NB28 were the only isolates that produced exudates on both SDA and PDA media at all three temperatures.

Aside from the variation among isolates, variation among repeats for the same isolate under the same conditions was also observed. Overall, of the 144 isolate-by-environment combinations, 79 showed no exudate production in any of the three repeats while 16, 20, and 29 showed exudate production in one, two and all three repeats respectively. In addition, the amount of exudate production also varied among many of the repeats (Fig. 3). For example, on SDA/14°C, two replicates produced observable exudates while one replicate did not produce any visible exudates at the time of our observation for each of the following three isolates NB7, ON16 and US3. In contrast, three isolates (NB1, ON3, and NS1) did not produce any exudates in any of the three replicates while the remaining 10 isolates each produced exudates in all three replicates.

### Pigment Secretion

**Figure 4:**
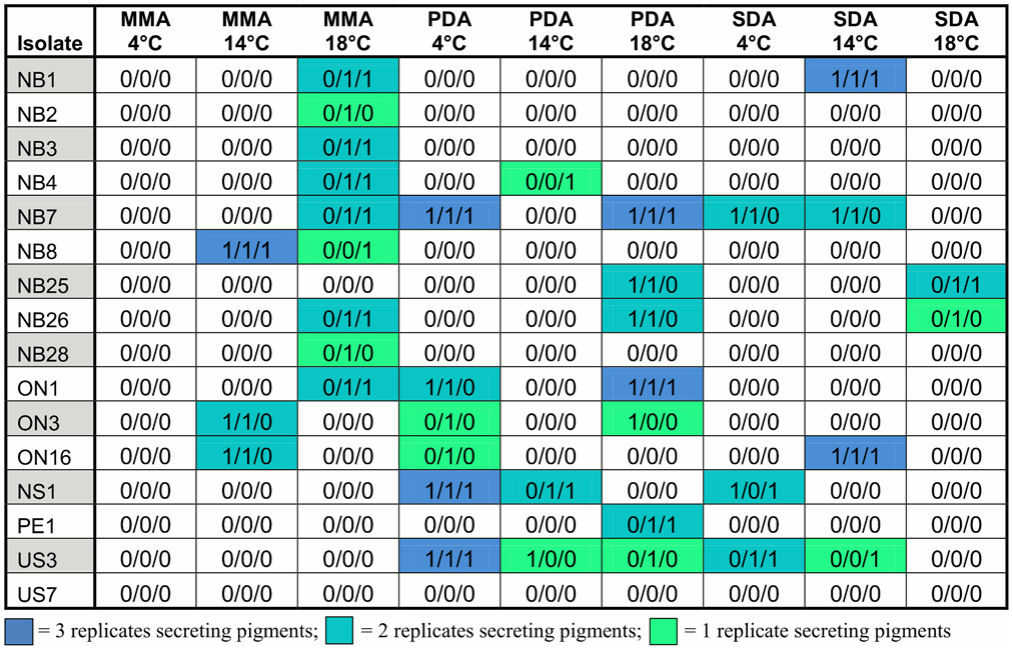
Variations in pigment diffusion within and among 16 North American *Pseudogymnoascus destructans* isolates in nine conditions. 0 = no visible pigment diffusion; 1 = visible pigment diffusion. SDA = Sabouraud dextrose agar, PDA = potato dextrose agar, MMA = minimal medium agar. Three replicates are shown for each medium-temperature condition for each isolate.

**Figure 5:**
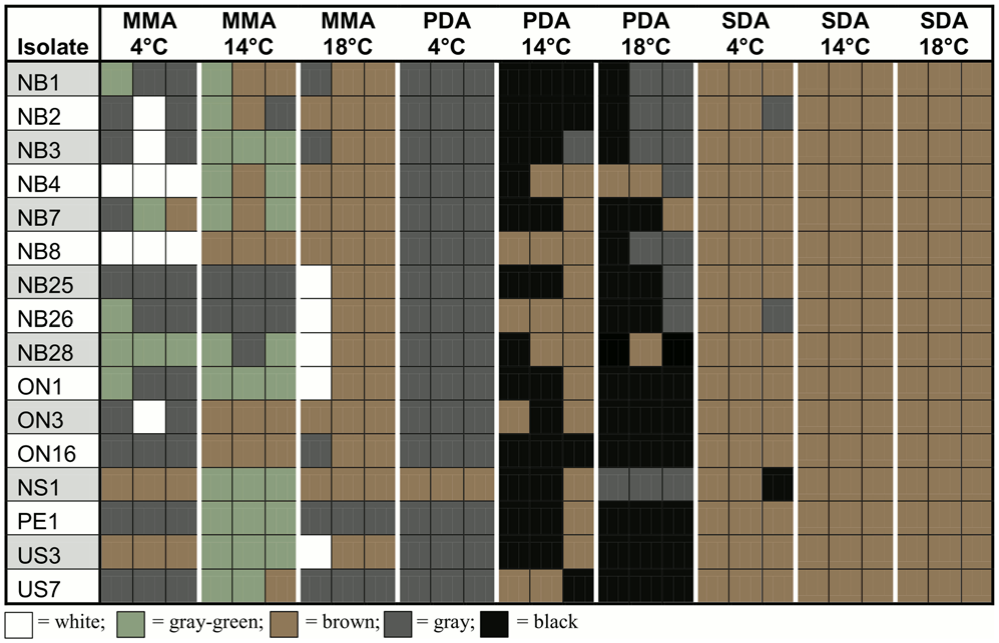
Variation in colony reverse color among 16 North American *Pseudogymnoascus destructans* isolates in nine conditions . SDA = Sabouraud dextrose agar, PDA = potato dextrose agar, MMA = minimal medium agar. Three replicates are shown for each medium-temperature condition for each isolate.

The secretion and diffusion of soluble pigments from the colony into the surrounding media was observed less frequently than exudate production (Fig. 4 and Fig. 5). Of the 144 isolate-environment combinations, 107 showed no pigment secretion and diffusion while 37 did in at least one of the three repeats. As with mycelial growth rate and exudate production, all three factors (isolate, medium, and incubation temperature) contributed to differences in pigment secretion and diffusion. Among the 16 isolates, one (US7) did not produce any diffusible pigment in any of the nine environments. Of the remaining 15 isolates, two (NB7 and US3) produced diffusible pigments in five conditions, five (NB26, ON1, ON3, ON16, and NS1) produced diffusible pigments in three conditions and four (NB1, NB4, NB8, and NB25) produced diffusible pigments in two conditions. The remaining four isolates (NB2, NB3, NB28, and PE1) produced diffusible pigments in only one condition (Fig. 4). Among the nine conditions, the number of isolates which produced diffusible pigments varied from 0 (MMA/4°C) to 9 (MMA/18°C), with both temperature and medium contributing to the observed differences (Fig. 4).

Similar to the observed variation among repeats for exudate production, variation in the production of diffusible pigments were also commonly seen among repeats for the same isolate in the same environmental condition. Among the 37 isolate-environment combinations that showed diffusible pigment production, 8 had all three repeats consistently producing diffusible pigments, while 18 had two repeats and 11 had one that produced diffusible pigments (Fig. 4). Figure 2A and 2C show a non-pigment secreting and a pigment secreting colony respectively.

### Colony Reverse Color

The color of the colony reverse seen from the bottom of the plates for all 16 isolates under the nine environmental conditions is presented in Figure 5. The observed color ranged from white to gray, gray-green, brown, and black. Similar to the other two qualitative traits examined for these isolates, all three factors (isolate, medium and incubation temperature) were found to influence the colony reverse color. For example, the colony reverse color was brown for all 16 isolates on SDA/14°C and SDA/18°C while only slight variations were found for three isolates on SDA/4°C. In contrast, the remaining two agar media showed a range of colors across these temperatures (Fig. 5). For example, on PDA/4°C, the colony reverse color was gray for all isolates except NS1, which was brown. However, on PDA/14°C, the colony reverse color was mostly brown to black. Compared to the relatively limited or no variation among isolates on SDA, the colony reverse colors among the isolates on MMA were more varied. For example, two isolates NB4 and NB8 were consistently white on MMA at the 4°C condition while other isolates produced a range of colors. Similar to the observed variation among replicates for exudate production and pigment diffusion, variation in colony reverse color were also found among replicates. For example, in the MMA/4°C condition, the colony reverse color of NB7 ranged between gray, gray-green and brown among replicates and in the PDA/18°C environment, colony reverse color of isolates ranged from brown to gray or gray to black among replicates. Figures 2E and 2F present a white reverse colony and a black reverse colony respectively.

## Discussion

Our analyses indicated that isolates of *P. destructans* from four provinces in eastern and central Canada all had the same multilocus sequence type, identical to that from the US. However, minor PCR fingerprinting variants were observed, consistent with micro- genotypic changes during the clonal spread of *P. destructans* in North America. In contrast to the limited genotype variation, abundant phenotypic variation was observed within and among 16 representative isolates. Furthermore, the pattern of phenotypic expression differed depending on environmental conditions. Below we discuss the potential mechanisms and implications of these observations.

### Clonal Expansion of a Single *P. destructans* Genotype in North America

Two hypotheses have been proposed to explain the emergence of an infectious disease [24]. The novel pathogen hypothesis suggests that a new disease emerges when a pathogen is introduced into a previously unoccupied geographical area and encounters a naïve host population. The endemic pathogen hypothesis suggests that changes in environmental factors or in the behaviour of the pathogen and/or host lead to disease emergence. Evidence that a single strain of *P. destructans* was introduced from Europe into North America suggests that the behavior of WNS in North America is best explained by the novel pathogen hypothesis [9]. The MLST data from our study showed 100% DNA sequence identity among *P. destructans* isolates in Canada and the US at the eight sequenced loci. This is consistent with the rapid clonal spread of a single genotype from a site or sites in the US to locations across much of eastern and central Canada.

Our PCR fingerprinting analyses revealed one dominant genotype for the majority of our 112 North American isolates. Three additional genotypes were also observed and each was represented by one isolate. All three unique genotypes originated from Canadian localities. As mentioned above, these three different genotypes were found to have a MLST genotype at the eight sequenced loci identical to those from sampled isolates originating from both Canada and the US. The presence of one dominant PCR fingerprint genotype from both the US and Canadian samples is consistent with the hypothesis of the clonal spread of a single *P. destructans* genotype throughout North America. However, the identification of three new PCR fingerprinting genotypes suggests that microevolution may be occurring or that there may be more natural genotypic variation in populations of *P. destructans* in North America than previously believed [11], [12]. It is important to note as well that the PCR fingerprinting methodology used in this study may have missed other types of genetic variation. A recent preliminary report based on data from whole-genome sequencing of multiple strains also suggested there was observable genetic variation among isolates of *P. destructans* in North America [25].

### Phenotypic Variation

In contrast to the observed MLST uniformity and minor PCR fingerprint genotype variation, we found significant phenotypic variation in all four examined traits within and among the 16 isolates that we screened. Given that very limited or no genotypic changes have been detected among North American *P. destructans* isolates using our methods, the large phenotypic differences within and among the isolates are surprising.

Rapid phenotypic diversification has been observed in laboratory conditions among replicate lines derived from a single ancestor, in a variety of microorganisms, due to the accumulation of spontaneous genetic mutations and the fixation of such mutations through random genetic drift [26]. *Glomus irregulare*, an arbuscular mycorrhizal fungus, produced significant genotypic and phenotypic variations over three generations among single spore lines derived from an initial spore [27]. However, their observed genotypic and phenotypic variations were different from what we found here. In *G. irregulare*, each spore is multinucleated and these nuclei can be genetically different. Through asexual reproduction and unequal nuclei partitioning, some progeny spore may inherit one nuclear genotype more frequently than the other(s), creating an asexual spore population with different spores having different proportions of different nuclei and such genetic differences can influence their phenotypes [27]. In *P. destructans*, none of the 44 sequenced isolates showed any heterozygosity at any of the eight sequenced loci and there was no evidence of genetically different nuclei residing in the same mycelia. Thus, in our case, the clonal progeny did not have different proportions of different nuclei. Even though minor genetic differences among isolates were found here and have been reported earlier [25], we did not find a clear relationship between the observed genetic and phenotypic variations among descendants of the North American *P. destructans* asexual lineage. Genome sequencing of a large number of isolates may be needed in order to identify the candidate genetic variants associated with the observed phenotypic variations.

There are a few possibilities to explain the phenotypic variation documented in this study. One is that there is little genetic basis for these three qualitative phenotypic traits and that these phenotypes are highly sensitive to minute environmental changes. Another is that the genome structures of many of the North American *P. destructans* isolates are not very stable and can change quickly, impacting phenotype expressions. The phenotypic traits examined in this study displayed phenotypic plasticity across the nine conditions and different isolates might vary in their plasticity. Other differences such as the thickness of the agar media and their relative order in the stack of agar plates could also contribute to the observed phenotypic variation among replicates of a single isolate. Furthermore, the within-isolate phenotypic variation might also be related to how the isolates were obtained. Specifically, isolates were obtained from swabs and purified on agar media through repeated sub-culturing and not obtained from single spore lines. Thus genetically different mycelia could exist in some of the isolates either from the original culture or from mutations accumulated during the culturing and sub-culturing in the lab, contributing to some of the phenotypic differences among replicates. Analyzing single spores from each isolate may reduce the phenotypic variation between replicates. Other possible causes for phenotypic variation could be that the thickness of the media affects the ability of pigments to diffuse through the media and be detected and the fluffiness of the aerial mycelia in some isolates may mask the extent of exudates produced. Finally, genetic mutations have likely accumulated during the clonal expansion of *P. destructans* in North America since 2006 that could have contributed to the among-strain differences and its phenotypic plasticity but our genotyping methods were unable to detect these genetic changes. Regardless of the potential mechanisms, the observed phenotypic diversity may have contributed to the rapid spread and high virulence of *P. destructans* in bats in North America. Below we discuss the potential significance for the four phenotypic traits investigated.

Colony diameter has been commonly used as an indicator of vegetative fitness in filamentous fungi [17]. The *P. destructans* mycelial growth rates between certain isolates differed by as much as two fold in several environmental conditions that we tested (Table S2). However, *in* *vitro* growth on artificial media may not reflect their mycelial growth in cave soil or *in* *vivo* in bats, as all of these substrata differ in their nutrient composition. Interestingly, two isolates obtained in this survey (NS1 & US3) demonstrated among the slowest growth rates but were also among those showing the most consistent pigment production and diffusion, and produced among the least exudates. These results suggest potential trade-offs among some of these traits in *P. destructans*. Specifically, slower growth rates and high pigment production might reflect a different strategy in nutrient allocation and utilization; where carbon and nitrogen resources could have been diverted from primary metabolism required for growth to secondary metabolism involved in pigment generation.

At present, the potential role(s) of exudate production in *P. destructans* is unknown. Exudates are commonly observed in fungal cultures *in* *vitro*. For example, many *Aspergillus* and *Penicillium* species have been characterized as producing exudate droplets, and this trait has been used for taxonomic differentiation between species within both genera [28]. Such droplets have been found to contain proteins and toxins. High levels of ochratoxins were reported in exudates in several *Penicillium* species [29]. A strain of *Aspergillus fumigatus* was reported to contain gliotoxin in its exudate [19]. *Metarhizium anisopliae*, an insect pathogen, contained destruxins and was shown to have protease-related enzyme activity in its exudate [20].

The ecological functions of exudates in nature are under debate. Jennings [30] believes that the exudates could act as a water reservoir for aerial hyphal growth in unfavourable conditions; whereas McPhee and Colotelo [31] suggest that exudate droplets might function as a reservoir of metabolic by-products, secondary metabolites, or metabolite reserves. The guttation droplets from *Fusarium culmorum* and *Sclerotinia sclerotiorum*, two plant fungal pathogens, have been shown to rapidly degrade plant tissue suggesting that exudate droplets may play a role in pathogenicity [32]. Whether exudates produced by *P. destructans* contain toxins or enzymes capable of damaging bats or degrading organic compounds in their surrounding environment remains to be investigated. However, studies have revealed that *P. destructans* can produce a variety of hydrolytic and proteolytic enzymes and utilize a diversity of carbon and nitrogen sources [12], [33]. Some of these proteolytic enzymes are implicated as virulence traits in a variety of pathogenic microbes [34]. The enzymes required to alter the surrounding environment of *P. destructans* may be exuded, permitting growth on various carbon sources and allowing different nitrogen sources to be used. The exudates may also act as a reservoir for secondary metabolites that aid hyphal growth. Interestingly, exudate production in our *P. destructans* isolates was only observed on PDA and SDA media; there was no exudate production from any of the isolates on MMA. Since the major difference between MMA and the other two media is the lack of free amino acids in MMA, the results suggest that the absence of a source of free amino acids may have a direct effect upon exudate production in *P. destructans*. The slightly higher pH of MMA (∼6.0) compared to SDA and PDA (5.6±0.2) could also affect the ability of *P. destructans* to produce exudates by altering enzymatic activity. However, in a previous study, the colony diameter of *P. destructans* was not significantly different across a wide pH range (5–11) indicative of its pH tolerance [33]. Therefore, we believe it’s unlikely that the slightly higher pH in MMA contributed to its lack of exudate production.

Both pigment production and diffusion varied significantly among isolates and among environmental conditions. While the nature of these pigments and their potential ecological roles are unknown, some of the pigments observed from the colony reverse are due to the melanisation of the mycelium, appearing as grey to brown or black in color. Most fungal melanins are produced from 1, 8-dihydroxynapthalene though the polyketide pathway or from 3–4 dihyroxyphenylalanine. In fungi, melanin deposition within the mycelia acts as a physical barrier to the external environment, filtering harmful UV radiation as well as protection against oxidative stress, and therefore imparts an enhanced survival and has been linked to virulence in several pathogenic species. For example, strains of *Cryptococcus neoformans* incapable of producing melanin were avirulent to mice [27], [28], [29], [35], whereas melanised isolates of the same species cause a disease state. Similarly, a mutant strain of *Aspergillus fumigatus* lacking conidial pigmentation also showed reduced virulence in mice [36]. Melanized strains of *C. neoformans* are less susceptible to nitrogen- and oxygen-derived oxidants, antibody-mediated phagocytosis and macrophages than non-melanized strains [18], [35], [36]. Furthermore, melanized *C. neoformans* were also more resistant to heat and cold, enzymatic degradation and UV light [37]–[39]. If the pigment in *P. destructans* plays a role similar to that demonstrated for melanin in other fungi and *in* *vitro* observations are consistent with hyphal melanisation *in* *vivo*, the significant variation among isolates in pigment production in *P. destructans* could contribute to differences in virulence or survival among the isolates.

## Conclusion

This study revealed limited genotypic variation among isolates of *P. destructans* from eastern and central Canada. Our results support the currently accepted hypothesis for a single recent introduction for the North American population of *P. destructans*. However, we found large differences in four phenotype traits examined, and that environmental factors significantly influenced the expression of these traits in laboratory culture. The genetic basis for the observed phenotypic differences and their ecological roles in *P. destructans* remain to be investigated.

## Supporting Information

Figure S1 Dendrogram showing the relationships among the 112 North American isolates of *Pseudogymnoascus destructans*. The dendrogram was created in PAUP 4.0 based on PCR fingerprinting patterns using primers (GACA)4 and M13 using the UPGMA method. Isolate WAC5705 of *P. pannorum* was used as an outgroup.
(TIF)

Table S1 Collection information of the 112 North American *Pseudogymnoascus destructans* isolates analyzed in this study. Highlighted in **bold** were those that were sequenced at the eight loci.
(DOCX)

Table S2 Mean colony diameter (n = 3; mm) at 28 days after inoculation of 16 representative North American *Pseudogymnoascus destructans* isolates grown on minimal medium agar (MMA), potato dextrose agar (PDA) and Sabouraud dextrose agar (SDA) in 4°C, 14°C and 18°C.
(DOCX)
