# Supplementary material for: Clonal Expansion of the Pseudogymnoascus destructans Genotype in North America Is Accompanied by Significant Variation in Phenotypic Expression
Source: PLoS One. 2014 Aug 14;9(8):e104684. doi: 10.1371/journal.pone.0104684 (PMC4133243; doi:10.1371/journal.pone.0104684)
Supplement: Table S1 — Collection information of the 112 North American Pseudogymnoascus destructans isolates analyzed in this study. Highlighted in bold were those that were sequenced at the eight loci. (DOCX) [file pone.0104684.s002.docx]

**Table S1.** Identification code with collection location and date for 112 North American *Pseudogymnoascus destructans* isolates sampled genetically and for phenotypic variation.

| **Isolate ID** | **Province/State** | **Location** | **Collection Date** |
| --- | --- | --- | --- |
| **NB1** | **New Brunswick** | **Markhamville Mine** | **2/23/2012** |
| **NB2** | **New Brunswick** | **Harbell’s Cave** | **2/28/2012** |
| **NB3** | **New Brunswick** | **Dorchester Mine** | **4/12/2012** |
| **NB4** | **New Brunswick** | **Glebe Mine** | **3/1/2012** |
| **NB5** | **New Brunswick** | **Harbell’s Cave** | **2/28/2012** |
| **NB6** | **New Brunswick** | **Markhamville Mine** | **3/1/2012** |
| **NB7** | **New Brunswick** | **Berryton Cave** | **3/6/2012** |
| **NB8** | **New Brunswick** | **White Cave** | **2/21/2012** |
| **NB9** | **New Brunswick** | **Glebe Mine** | **4/10/2012** |
| **NB10** | **New Brunswick** | **Berryton Cave** | **3/6/2012** |
| NB11 | New Brunswick | Berryton Cave | 3/6/2012 |
| **NB12** | **New Brunswick** | **Berryton Cave** | **3/6/2012** |
| NB13 | New Brunswick | Berryton Cave | 3/6/2012 |
| NB14 | New Brunswick | Berryton Cave | 3/6/2012 |
| **NB15** | **New Brunswick** | **White Cave** | **2/21/2012** |
| **NB16** | **New Brunswick** | **Berryton Cave** | **4/6/2012** |
| NB17 | New Brunswick | Berryton Cave | 4/6/2012 |
| NB18 | New Brunswick | Berryton Cave | 3/6/2012 |
| **NB19** | **New Brunswick** | **Berryton Cave** | **3/6/2012** |
| NB20 | New Brunswick | Berryton Cave | 3/6/2012 |
| NB21 | New Brunswick | Berryton Cave | 3/6/2012 |
| **NB22** | **New Brunswick** | **Berryton Cave** | **2011** |
| **NB23** | **New Brunswick** | **Glebe Mine** | **4/10/2012** |

| **NB24** | **New Brunswick** | **Markhamville Mine** | **2/23/2012** |  |
| --- | --- | --- | --- | --- |
| **NB25** | **New Brunswick** | **Howes Cave** | **2/24/2012** |  |
| **NB26** | **New Brunswick** | **Dorchester Mine** | **4/12/2012** |  |
| NB27 | New Brunswick | Dorchester Mine | 4/12/2012 |  |
| **NB28** | **New Brunswick** | **Harbell’s Cave** | **2/28/2012** |  |
| NB29 | New Brunswick | White Cave | 2/21/2012 |  |
| NB30 | New Brunswick | Markhamville Mine | 3/1/2012 |  |
| NB31 | New Brunswick | Harbell’s Cave | 2/28/2012 |  |
| NB32 | New Brunswick | Markhamville Mine | 2/23/2012 |  |
| NB33 | New Brunswick | Glebe Mine | 3/1/2012 |  |
| NB34 | New Brunswick | White Cave | 2/21/2012 |  |
| NB35 | New Brunswick | Dorchester Mine | 4/12/2012 |  |
| NB36 | New Brunswick | Harbell’s Cave | 2/28/2012 |  |
| NB37 | New Brunswick | Harbell’s Cave | 2/28/2012 |  |
| NB38 | New Brunswick | Markhamville Mine | 3/1/2012 |  |
| NB39 | New Brunswick | Markhamville Mine | 2/23/2012 |  |
| NB40 | New Brunswick | Markhamville Mine | 3/1/2012 |  |
| NB41 | New Brunswick | Glebe Mine | 4/10/2012 |  |
| NB42 | New Brunswick | White Cave | 2/21/2012 |  |
| NB43 | New Brunswick | Markhamville Mine | 3/1/2012 |  |
| NB44 | New Brunswick | Glebe Mine | 4/10/2012 |  |
| NB45 | New Brunswick | White Cave | 2/21/2012 |  |
| NB46 | New Brunswick | White Cave | 2/21/2012 |  |
| NB47 | New Brunswick | Glebe Mine | 4/10/2012 |  |
| NB48 | New Brunswick | Dorchester Mine | 4/12/2012 |  |
| NB49 | New Brunswick | Dorchester Mine | 4/12/2012 |  |
| NB50 | New Brunswick | Glebe Mine | 4/10/2012 |  |
| NB51 | New Brunswick | Harbell’s Cave | 2/28/2012 |  |
| NB52 | New Brunswick | White Cave | 2/21/2012 |  |
| NB53 | New Brunswick | Berryton Cave | 4/6/2012 |  |
| NB54 | New Brunswick | White Cave | 2/21/2012 |  |
| NB55 | New Brunswick | Markhamville Mine | 3/1/2012 |  |
| NB56 | New Brunswick | White Cave | 2/21/2012 |  |
| NB57 | New Brunswick | Glebe Mine | 3/1/2012 | |
| NB58 | New Brunswick | White Cave | 2/21/2012 | |
| NB59 | New Brunswick | White Cave | 2/21/2012 | |
| NB60 | New Brunswick | White Cave | 2/21/2012 | |
| NB61 | New Brunswick | Glebe Mine | 4/10/2012 | |
| NB62 | New Brunswick | Markhamville Mine | 2/23/2012 | |
| NB63 | New Brunswick | Harbell’s Cave | 2/28/2012 | |
| NB64 | New Brunswick | Markhamville Mine | 3/1/2012 | |
| NB65 | New Brunswick | White Cave | 2/21/2012 | |
| NB66 | New Brunswick | Markhamville Mine | 3/1/2012 | |
| NB67 | New Brunswick | Markhamville Mine | 2/23/2012 | |
| NB68 | New Brunswick | White Cave | 2/21/2012 | |
| NB69 | New Brunswick | Markhamville Mine | 2/23/2012 | |
| NB70 | New Brunswick | White Cave | 2/21/2012 | |
| NB71 | New Brunswick | White Cave | 2/21/2012 | |
| **PE1** | **Prince Edward Island** | **Rocky Point, Queens County** | **3/3/2013** | |
| PE2 | Prince Edward Island | Rocky Point, Queens County | 3/3/2013 | |
| **PE3** | **Prince Edward Island** | **Uigg, Queens County** | **3/8/2013** | |
| **PE4** | **Prince Edward Island** | **Vernon Bridge, Queens County** | **3/14/2013** | |
| **PE5** | **Prince Edward Island** | **Prim Point, Queens County** | **3/18/2013** | |
| **PE6** | **Prince Edward Island** | **Caledonia, Queens County** | **4/10/2013** | |
| **PE7A** | **Prince Edward Island** | **Murray River, Kings County** | **4/15/2013** | |
| PE7B | Prince Edward Island | Murray River, Kings County | 4/15/2013 | |
| **PE9** | **Prince Edward Island** | **Panmure Island, Kings County** | **4/23/2013** | |
| **NS1** | **Nova Scotia** | **Falmouth, Hants County** | **4/4/2013** | |
| **ON1** | **Ontario** |  |  | |
| **ON2** | **Ontario** |  |  | |
| **ON3** | **Ontario** |  |  | |
| **ON4** | **Ontario** |  |  | |
| **ON5** | **Ontario** |  |  | |
| **ON6** | **Ontario** |  |  | |
| **ON7** | **Ontario** |  |  | |
| **ON8** | **Ontario** |  |  | |
| **ON9** | **Ontario** |  |  | |
| **ON10** | **Ontario** |  |  | |
| **ON11** | **Ontario** |  |  | |
| **ON12** | **Ontario** |  |  | |
| **ON13** | **Ontario** |  |  | |
| **ON14** | **Ontario** | **Rattlesnake Point, Milton** | **3/16/2012** | |
| **ON15** | **Ontario** |  |  | |
| **ON16** | **Ontario** |  |  | |
| US1 | Vermont | Greely Mine | 2013 | |
| US2 | Vermont | Greely Mine | 2013 | |
| US3 | New York | Williams Lake Mine | 2013 | |
| US4 | New York | Williams Lake Mine | 2013 | |
| US5 | West Virginia | Greenbrier | 12/3/2010 | |
| US6 | Pennsylvania | Woodward | 3/25/2010 | |
| US7 | North Carolina | Avery | 3/2/2011 | |
| US8 | North Carolina | Avery | 3/2/2011 | |
| US9 | West Virginia | Tucker | 3/23/2011 | |
| US10 | North Carolina | Yancey | 8/2/2011 | |
| US11 | Ohio | Lawrence | 3/22/2011 | |
| US12 | West Virginia | Fayette | 3/23/2011 | |
| US13 | West Virginia | Randolph | 11/3/2011 | |
| US14 | West Virginia | Pendleton | 3/23/2011 | |
| US15 | New York | Williams Hotel Mine | 3/28/2008 | |

Bolded isolates were sequenced at the eight genetic loci.
