# Supplementary material for: Clonal Expansion of the Pseudogymnoascus destructans Genotype in North America Is Accompanied by Significant Variation in Phenotypic Expression
Source: PLoS One. 2014 Aug 14;9(8):e104684. doi: 10.1371/journal.pone.0104684 (PMC4133243; doi:10.1371/journal.pone.0104684)
Supplement: Table S2 — Mean colony diameter (n = 3; mm) at 28 days after inoculation of 16 representative North American Pseudogymnoascus destructans isolates grown on minimal medium agar (MMA), potato dextrose agar (PDA) and Sabouraud dextrose agar (SDA) in 4°C, 14°C and 18°C. (DOCX) [file pone.0104684.s003.docx]

**Table S2.** Mean colony diameter (n=3; mm) 28 days after inoculation of 16 North American *Pseudogymnoascus destructans* isolates grown on minimal media (MM), potato dextrose agar (PDA) and synthetic-dextrose (SD) in 4°C, 14°C and 18°C.

|  | **MM 4°C^2^** | **MM 14°C^1^** | **MM 18°C^2^** | **PDA 4°C^2^** | **PDA 14°C^2^** | **PDA 18°C^1^** | **SD 4°C^1^** | **SD 14°C^2^** | **SD 18°C^1^** |
| --- | --- | --- | --- | --- | --- | --- | --- | --- | --- |
| NB1 | 9.3 ± 1.5 (abc)^3^ | 14.3 ± 2.1 (abef) | 8.0 ± 1.0 (ab) | 14.3 ± 1.2 (abc) | 21.7 ± 0.6 (d) | 15.0 ± 1.7 (a) | 14.8 ± 1.3 (e) | 22.7 ± 0.6 (c) | 9.7 ± 0.6 (ab) |
| NB2 | 8.0 ± 0.0 (abc) | 15.7 ± 2.5 (ade) | 7.2 ± 1.3 (ab) | 13.3 ± 1.5 (abc) | 19.3 ± 0.6 (ade) | 14.0 ± 1.7 (ab) | 13.2 ± 0.8 (adef) | 19.3 ± 0.6 (ade) | 9.5 ± 0.9 (ab) |
| NB3 | 8.0 ± 0.0 (abc) | 15.7 ± 1.2 (ade) | 7.5 ± 0.5 (a) | 12.7 ± 1.2 (abc) | 20.3 ± 0.6 (ad) | 14.0 ± 1.0 (ab) | 13.7 ± 1.5 (aef) | 21.3 ± 1.2 (acd) | 9.3 ± 1.2 (ab) |
| NB4 | 12.0 ± 1.0 (ac) | 14.7 ± 0.6 (abe) | 8.0 ± 0.0 (ac) | 12.0 ± 0.0 (ac) | 21.0 ± 1.0 (ade) | 12.3 ± 1.5 (ab) | 14.0 ± 1.0 (ae) | 20.7 ± 0.6 (acd) | 9.7 ± 0.6 (ab) |
| NB7 | 8.0 ± 0.0 (abc) | 15.7 ± 1.2 (ade) | 7.2 ± 0.8 (ab) | 10.7 ± 0.6 (ab) | 18.3 ± 0.6 (ae) | 13.3 ± 2.1 (ab) | 10.7 ± 1.2 (fg) | 20.0 ± 1.0 (acde) | 7.7 ± 1.5 (a) |
| NB8 | 9.8 ± 0.3 (c) | 20.7 ± 2.1 (d) | 7.7 ± 0.6 (a) | 12.7 ± 0.6 (ac) | 20.3 ± 0.6 (ad) | 14.0 ± 1.7 (ab) | 12.5 ± 0.9 (acdef) | 21.7 ± 0.6 (cd) | 9.5 ± 1.5 (ab) |
| NB25 | 8.3 ± 0.6 (abc) | 15.3 ± 0.6 (ae) | 6.7 ± 0.6 (ab) | 12.7 ± 1.2 (abc) | 19.7 ± 0.6 (ad) | 13.3 ± 1.2 (ab) | 13.3 ± 1.5 (aef) | 21.3 ± 0.6 (acd) | 9.7 ± 1.5 (ab) |
| NB26 | 10.0 ± 1.0 (acd) | 15.7 ± 1.5 (ade) | 7.7 ± 0.6 (a) | 13.7 ± 0.6 (c) | 20.3 ± 1.2 (acde) | 14.3 ± 0.6 (a) | 13.7 ± 0.6 (aef) | 21.7 ± 0.6 (cd) | 9.5 ± 1.8 (ab) |
| NB28 | 9.7 ± 1.5 (abc) | 17.3 ± 2.1 (ad) | 7.8 ± 0.3 (a) | 14.0 ± 0.0 (ac) | 18.7 ± 0.6 (ae) | 13.8 ± 1.9 (ab) | 14.3 ± 1.2 (ae) | 22.0 ± 1.0 (acd) | 11.3 ± 1.4 (b) |
| ON1 | 7.3 ± 0.6 (abc) | 12.0 ± 1.0 (ef) | 6.7 ± 1.5 (ab) | 10.0 ± 0.0 (abc) | 17.3 ± 2.1 (abde) | 13.0 ± 1.0 (ab) | 10.0 ± 0.0 (bdg) | 18.7 ± 0.6 (ae) | 9.0 ± 1.0 (ab) |
| ON3 | 9.0 ± 1.0 (abc) | 16.7 ± 2.1 (ade) | 6.0 ± 0.0 (ab) | 12.0 ± 1.0 (abc) | 19.7 ± 1.5 (abde) | 13.7 ± 0.6 (ab) | 11.0 ± 1.0 (abg) | 18.0 ± 1.0 (acde) | 8.3 ± 1.2 (ab) |
| ON16 | 8.2 ± 0.3 (ab) | 15.2 ± 2.8 (abe) | 5.7 ± 0.6 (ab) | 11.8 ± 1.0 (abc) | 16.3 ± 1.5 (abde) | 13.3 ± 1.2 (ab) | 12.0 ± 1.0 (abef) | 18.7 ± 0.6 (ae) | 9.3 ± 1.2 (ab) |
| NS1 | 5.2 ± 0.8 (b) | 10.0 ± 0.0 (bcf) | 4.2 ± 0.3 (b) | 8.5 ± 0.5 (b) | 14.0 ± 0.0 (bcf) | 10.0 ± 1.0 (b) | 9.3 ± 1.2 (bcg) | 15.0 ± 1.0 (be) | 6.7 ± 0.6 (a) |
| PE1 | 8.7 ± 1.5 (abc) | 18.7 ± 1.5 (ad) | 7.3 ± 1.2 (ab) | 12.3 ± 0.6 (ac) | 18.0 ± 1.0 (acde) | 14.3 ± 1.2 (a) | 13.3 ± 1.5 (aef) | 19.7 ± 0.6 (ade) | 10.0 ± 1.7 (ab) |
| US3 | 6.3 ± 0.6 (bd) | 9.2 ± 0.3 (cf) | 4.7 ± 0.6 (bc) | 10.2 ± 0.7 (abc) | 11.0 ± 0.0 (b) | 11.0 ± 1.0 (ab) | 9.0 ± 1.0 (bg) | 12.3 ± 1.2 (b) | 7.7 ± 0.6 (a) |
| US7 | 8.0 ± 1.0 (abc) | 15.3 ± 2.5 (ae) | 5.7 ± 0.6 (ab) | 12.3 ± 0.6 (ac) | 16.7 ± 0.6 (ef) | 12.7 ± 0.6 (ab) | 14.8 ± 0.3 (ae) | 17.3 ± 2.9 (abc) | 10.0 ± 1.0 (ab) |
| Average | 8.5 ± 1.7 | 15.1 ± 3.1 | 6.7 ± 1.3 | 12.1 ± 1.7 | 18.3 ± 2.8 | 13.3 ± 1.7 | 12.5 ± 2.1 | 19.4 ± 2.8 | 9.2 ± 1.5 |
| Significant Pairs (out of 120 pairs total) | 6 | 30 | 9 | 7 | 25 | 3 | 34 | 28 | 3 |

**^1^** Tukey’s Honest Significant Difference Test

**^2^** Games-Howell test

^3,^ Different letters denote significant differences between isolates within each of the nine environmental conditions (p <0.05). Specifically, if two isolates shared any letter between them (e.g. between one with "abc" and another with "bd" as they share the letter "b"), it denoted that they were not significantly different from each other. On the other hand, if two isolates shared no letter (e.g. between one with "abc" and another with "de"), then they are statistically different in their growth rates at the specific condition. Data are presented as mean ± SD.
